# COVID-19 Is Associated With an Increase in Visits for Anxiety but Not Depression

**DOI:** 10.7759/cureus.17789

**Published:** 2021-09-07

**Authors:** Elizabeth Pfoh, Kathryn A Martinez, Jessica Hohman, Anita Misra-Hebert, Michael Rothberg

**Affiliations:** 1 Center for Value-Based Care Research, Cleveland Clinic, Cleveland, USA; 2 Cleveland Clinic Community Care, Cleveland Clinic, Cleveland, USA

**Keywords:** depression, anxiety, primary care, covid-19, mental health disorders

## Abstract

Background

The coronavirus disease 2019 (COVID-19) pandemic has increased concerns about mental health. We conducted a time-series analysis to determine whether the percentage of primary care visits for anxiety and depression changed after COVID-19.

Methodology

We assessed the adjusted weekly change in the percentage of primary care visits for anxiety and depression between August 2019 and October 2020 at a large integrated health system. To account for changes in overall visit behavior during the pandemic, we created three periods: pre-period (August 1, 2019 to March 8, 2020), initial period (March 9, 2020 to June 31, 2020), and return period (July 1, 2020 to October 31, 2020). We used hierarchical linear regression models (clustered by month) to identify the association between the time period and the adjusted mean weekly percentage of visits for depression or anxiety. We conducted the analysis in 2020 and 2021.

Results

There were 1,691,071 encounters among 605,105 unique adults. The median age was 55 years (interquartile range = 39-68), 57% were female, 78% were white, and 59% had private insurance. Most visits were office-based (versus virtual), of which 99% were in the pre-COVID-19 period and 75% in the return period. There was a significant increase in the percentage of visits associated with anxiety after July compared to before COVID-19 (10.4% versus 9.2%; p = 0.006), and there was no difference in the percentage of visits for depression (p > 0.05).

Conclusions

Outreach to individuals with depression who have not sought care may be necessary.

## Introduction

The coronavirus disease 2019 (COVID-19) pandemic has led to concern about the deterioration of mental health [1,2]. In August 2020, the Centers for Disease Control and Prevention (CDC) reported that anxiety symptoms had increased three-fold and depression symptoms had increased four-fold compared to 2019 [3]. In addition, symptoms of depression and anxiety have remained elevated throughout the fall of 2020 [4]. Both conditions are frequently treated in primary care [5]. However, how primary care was delivered changed during the early part of the pandemic, potentially prompting differences in how patients sought care [6].

Given the robust epidemiological evidence that symptoms of depression and anxiety increased, it is important to understand whether patients are accessing care for their mental health needs. Hence, the objective of this study was to determine whether the percentage of primary care visits for anxiety and depression changed after the start of the COVID-19 pandemic.

## Materials and methods

Our time series analysis included adults (>18 years of age) with a primary care visit between August 2019 and October 2020 at a large integrated health system in Ohio. We included both in-person and virtual (telephone/video) visits within internal medicine or family medicine practices. We excluded urgent care visits. Based on the International Classification of Diseases, Tenth Revision codes, we identified visits for depression (codes F32, F33, F54) or anxiety (codes F41, F43). If a patient discussed both depression and anxiety during the visit, then the visit was coded for both diseases. Cleveland Clinic’s Institutional Review Board approved this study.

To account for changes in the overall visit behavior during the pandemic, we created three periods reflective of Ohio’s COVID-19 response: pre-period (August 1, 2019 to March 8, 2020), initial period (March 9, 2020 to June 31, 2020), and return period (July 1, 2020 to October 31, 2020) [7]. During the initial period, patients were urged to use electronic visits (versus in-person) visits. During the return period, patients were able to choose whether they wanted to have an in-person or electronic visit.

Subsequently, we separately assessed the weekly change in the percentage of visits for anxiety and depression. We chose the percentage of visits as our outcome because the total visit volume dropped at the beginning of the pandemic and then recovered.

Analysis

For each week of the study period, we identified the average percentage of primary care visits for anxiety or depression after adjusting for age, sex, race, and insurance status of the patients. We used hierarchical linear regression models to identify the association between the time period and the adjusted mean weekly percentage of visits for depression or anxiety. Time in months was included as a random effect. Results were identified as significant at P-values of <0.05 using two-sided significance tests.

We conducted a secondary analysis to identify if the overall findings were similar among subpopulations (white versus black individuals; female versus male individuals). For this, we used a hierarchical linear regression model to identify the association between race or sex and the adjusted percentage of visits for depression or anxiety. We included the interaction between the period of time and the subpopulation as a fixed variable and patient identifier and time as random variables. Finally, to identify whether our main findings were due to changes in other visit types, we conducted a sensitivity analysis using the number of visits as the outcome. We conducted the analysis using Stata version 14.0 (StataCorp LLC, College Station, TX) in 2020 and 2021.

## Results

There were 1,691,071 encounters among 605,105 unique adults. The median age was 55 years (interquartile range = 39-68), 57% were female, 78% were white, and 59% had private insurance. Most visits were office-based (versus virtual), of which 99% were in the pre-COVID-19 period and 75% were in the return period.

**Figure 1:**
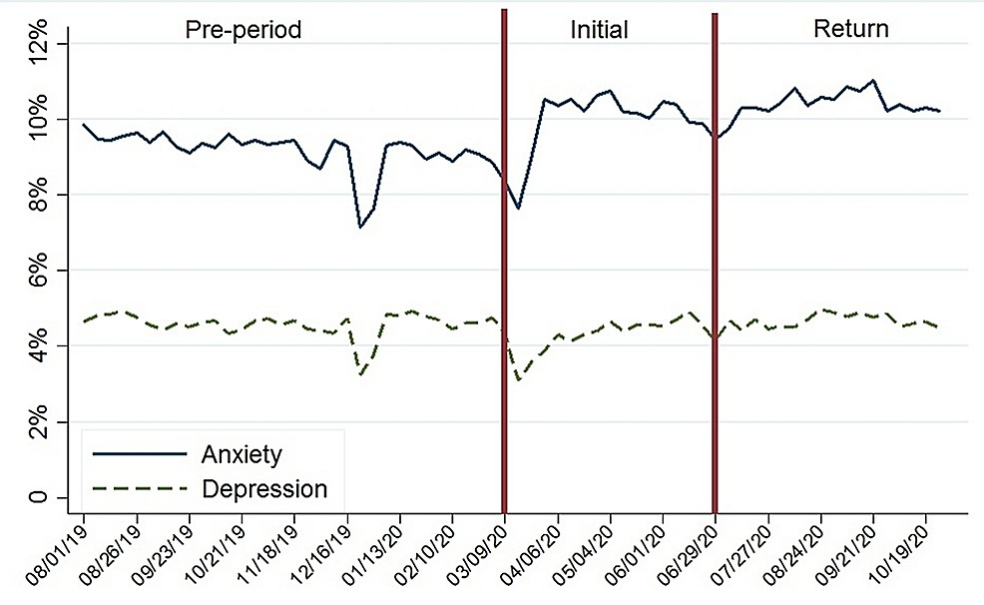
Adjusted weekly percentage of visits for depression or anxiety between August 2019 and October 2020.

Figure 1 shows the adjusted percentage of visits for anxiety and depression by time period. In the initial period, there was an increase in the percentage of visits associated with anxiety versus pre-period (9.9% versus 9.2%; p < 0.001); yet, for depression, the percentage of visits decreased slightly over this time period versus pre-period (4.2% versus 4.6%; p = 0.008). In the return period, there was a significant increase in the adjusted percentage of visits associated with anxiety versus the pre-period (10.4% versus 9.2%; p = 0.006), and there was no difference in the percentage of visits for depression (p > 0.05).

In our secondary analysis, a lower percentage of visits among black adults were for anxiety (β = -5.1% [95% confidence interval (CI) = -5.1%, -5.1%]) and a slightly higher percentage of visits were for depression (β = 0.3% [95% CI = 0.3%, 0.3%]) than white adults. Similar to the main analysis, the percentage of visits for anxiety among black adults increased in the return period compared to the pre-period (β = 5.5% [95% CI = 5.4%, 5.4%] in the pre-period and β = 5.8% [95% CI = 5.8%, 5.8%] in the return period). The percentage of visits for depression was similar among black adults in the return period and the pre-period (β = 4.8% [95% CI = 4.8%, 4.8%] in the pre-period versus β = 4.8% [95% CI = 4.8%, 4.8%] in the return period). Additionally, compared to females, a slightly lower percentage of visits by males were for depression (β = -0.04% [95% CI = -0.05%, -0.04%]) and a similar percentage of visits were for anxiety (p > 0.05).

In our sensitivity analysis, the number of anxiety visits per week increased in the return period compared to the pre-period (β = 220 visits; p = 0.04) and decreased in the initial period compared to the pre-period (β = -323 visits; p = 0.002). The number of visits per week for depression decreased in the initial period (β = 299 visits; p < 0.001) compared to the pre-period, and there was no difference in the return period compared to the pre-period (p > 0.05). All visits decreased in the initial period compared to the pre-period (p < 0.01) and were similar in the return period (p > 0.05).

## Discussion

Following the initial phase of the COVID-19 shutdown, we found an increase in the percentage of visits for anxiety but not depression. This was surprising because, based on prior survey data [3,4], we expected to find an increase in visits for both conditions.

The increase in visits for anxiety could represent a higher prevalence or more severe anxiety prompting treatment-seeking behavior. A survey of primary care patients in Hong Kong between March and April 2020 found that anxiety symptoms increased compared to the year earlier, but not depression [8]. Depression likely increased based on the CDC’s Household Pulse survey results [3] but may have gone untreated. This may be due to less severe symptoms, barriers to access to care, or the inability to seek care due to the depression itself. While we cannot determine why there was not the expected increase in visits for depression, this information is useful for future research and operational initiatives.

Our sensitivity analysis found that the number of visits for depression and anxiety decreased in the initial period. This fits with the current literature showing a decrease in primary care visits in the initial period of the COVID-19 pandemic [9,10]. We also found that while the number of visits for depression was similar in the return period compared to the pre-period, the number of visits for anxiety was higher. Understanding the changes in visits for depression and anxiety in primary care is particularly important as most patients with a mental illness consult a primary care physician [11].

Due to isolation and uncertainty induced by COVID-19, there is a concern about an increase in deaths of despair; and in this setting, it is concerning that we did not see a higher percentage of visits for depression. Fortunately, suicides have not increased during periods of lock-down in the United States [12], suggesting that the increase in reporting on surveys may not represent severe depression. Such individuals may recover without treatment. A more concerning scenario would be if individuals in distress go untreated due to inability to access care.

Our study had the following limitations. First, data were from a single health system representing only visit diagnosis codes in primary care. Second, it is impossible to assign reasons for the increase in one diagnosis but not the other. Third, there may have been a seasonality effect in the data that we could not account for due to the limited time period. Fourth, we did not have a measure of depression or anxiety severity such as the Patient Health Questionnaire-9 or the General Anxiety Disorder-7.

## Conclusions

In the six months following the onset of the COVID-19 pandemic, primary care visits increased for anxiety but not for depression. This may be due to patients with symptoms of depression not accessing care. Outreach to vulnerable patients may be necessary to identify those with depression who have not sought care. In addition, policymakers should continue to support systems that make accessing care easier for patients, such as electronic visits.
